# Fish Tank Granuloma Presenting as a Nasal Cavity Mass

**DOI:** 10.1155/2021/8820720

**Published:** 2021-01-12

**Authors:** Motoki Sekine, Fumiyuki Goto, Kosuke Saito, Shoji Kaneda, Hikaru Yamamoto, Tomoaki Murakami, Takahide Hamano, Kenji Okami

**Affiliations:** Department of Otolaryngology, Tokai University School of Medicine, 143 Shimokasuya, Isehara, Kanagawa 259-1193, Japan

## Abstract

*Mycobacterium marinum* is a free-living nontuberculous mycobacterium that is widely distributed in freshwater and seawater around the world. Granulomatous skin infection from *M. marinum* in people who are exposed to fish or aquatic environments is a rare condition known as fish tank granuloma. The granuloma mainly occurs on the skin of the upper limb, in a few cases on the face, and rarely in the nasal cavity. We describe a case of *M. marinum* infection that presented as a nasal cavity mass. A 57-year-old woman who was receiving infliximab for psoriatic arthritis visited our hospital with a complaint of right nasal obstruction. A granulomatous mass with an irregular surface was found in the anterior part of the right nasal cavity. Tissue biopsy revealed granulation tissue. Since the application of steroid ointment did not reduce the size of the mass, the tumor was resected under local anesthesia, and the base was cauterized. The pathological finding was an inflammatory granuloma with negative Ziehl–Neelsen staining. The granuloma recurred 3 months after resection. The interferon-gamma release assay (IGRA) test was positive, and therefore, a mycobacterial tissue culture test was performed because of suspected nasal tuberculosis, which identified *M. marinum*. The nasal cavity mass disappeared 2 months after the administration of minocycline, followed by clarithromycin, and subsequent discontinuation of infliximab. *M. marinum* infection can cause an intranasal mass. IGRA and the mycobacterial tissue culture test are useful for diagnosis. As in this case, the nasal lesion may be excised as an inflammatory nasal granuloma, and therefore, there may be many more “hidden” cases of *M. marinum* infection. If nasal granulation is present, the possibility of *M. marinum* infection should be considered.

## 1. Introduction

Infections by nontuberculous mycobacteria are increasing with the increase in the number of patients who use immunosuppressive agents, including antitumor necrosis factor (anti-TNF) biologics [1, 2]. *Mycobacterium marinum*, which mainly lives in water, accounts for the majority of nontuberculous mycobacteria infections occurring in the skin. In the United States, the incidence of cutaneous nontuberculous mycobacterial infection between 1990 and 2009 was 1.3 per 100,000, of which 45% were *M. marinum* infections [3]. Fish tank granuloma is an infection by *M. marinum* that causes a mass or ulcer on the skin of people who are exposed to aquatic environments including fish tank water [4]. Ninety-three percent of *M. marinum* infections occur on the upper limbs, and a few occur on the face [5]. Only a few cases of *M. marinum* infections have been reported as intranasal lesions [6, 7]. We report a case of *M. marinum* infection that presented as a nasal cavity mass. Interferon-gamma release assay (IGRA) was important for diagnosis. This paper is a rare case report of this infection in the nasal cavity.

The limitation of this study is that this is just a single case report, and the detailed epidemiological date including prevalence of the disease in the nasal cavity is not clearly identified. It should be done for confirmation in the future epidemiologic studies.

## 2. Case Presentation

**Figure 1:**
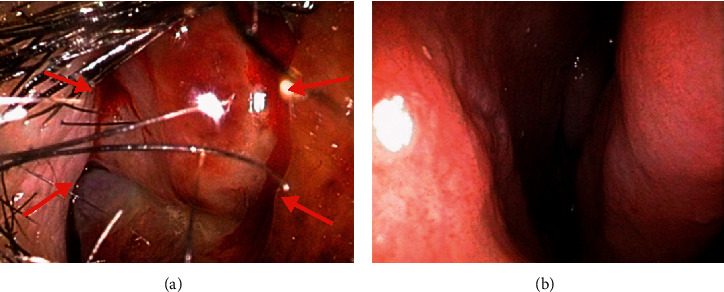
Endoscopic nasal examination revealed a granulomatous mass with an irregular surface in the anterior right nasal cavity (arrow). (a) Right side. (b) Left side.

**Figure 2:**
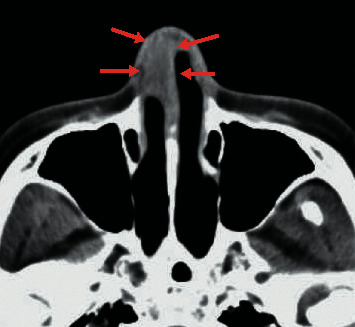
Computed tomography scan revealed a mass lesion localized in the anterior part of the right nasal cavity (arrow).

**Figure 3:**
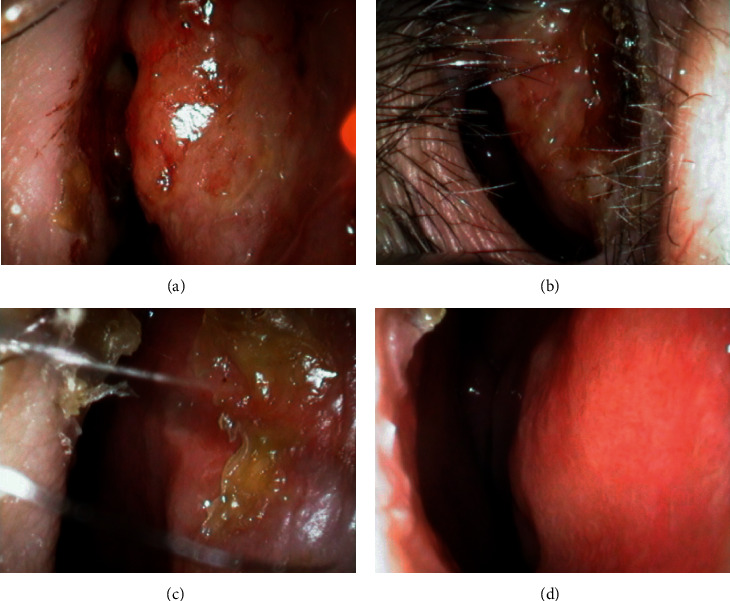
Complete resolution of lesions occurred in response to 4 weeks of oral minocycline (100 mg twice daily) followed by 4 weeks of clarithromycin (200 mg twice daily) and discontinuation of infliximab. (a) 2 weeks later. (b) 4 weeks later. (c) 6 weeks later. (d) 8 weeks later.

A 57-year-old woman had received infliximab for psoriatic arthritis for 5 years. She was admitted to our hospital with a complaint of right nasal obstruction for 1 month. A granulomatous mass with an irregular surface was found in the anterior right nasal cavity (Figure 1). A computed tomography scan revealed a tumor shadow localized in front of the right nasal cavity (Figure 2). A tissue sample was obtained with nasal forceps, and histological examination showed granulation tissue. Application of steroid ointment did not reduce the size of the tumor, and therefore, the tumor was resected under local anesthesia 6 months after the first visit. The tumor was attached to the anterior nasal septum, and after excision, the base was cauterized. Pathological examination revealed an epithelioid granuloma with multinucleated giant cells. Ziehl–Neelsen staining was negative. The tumor recurred 3 months after resection. IGRA (T-SPOT.TB test) was positive, and we performed tissue culture for mycobacteria because of suspected nasal tuberculosis. The smear microscopy was grade 1, and the polymer chain reaction tests for tuberculosis and *Mycobacterium avium* complex were negative. Individual orange-yellow colonies developed after 8 weeks of incubation on liquid (MGIT) media at 37°C and after 3 weeks of incubation on 2% Ogawa egg medium at 30°C. *M. marinum* was identified using matrix-assisted laser desorption/ionization-time-of-flight mass spectrometry. The lesion disappeared 2 months after administration of oral minocycline (4 weeks, 100 mg twice daily) followed by oral clarithromycin (4 weeks, 200 mg twice daily) and discontinuation of infliximab (Figure 3). After the clinical diagnosis was confirmed, a detailed life history was taken. The life history revealed that she has many tropical fish and sometimes uses her bare hands to clean fish tanks.

The patient provided written consent for the use of her clinical details in this case report. The study was conducted in accordance with the Declaration of Helsinki (1964).

## 3. Discussion

The course of this patient resulted in two important clinical suggestions: *M. marinum* infection can cause an intranasal mass, and IGRA and the mycobacterial tissue culture test are useful for diagnosis.

First, *M. marinum* infection can cause an intranasal mass. According to a recent review [5], 93% of *M. marinum* infections occur in the upper limbs, and there are few facial lesions. Only a few cases have been reported as intranasal lesions [6, 7]. Since *M. marinum* exists widely in freshwater and seawater, the infection often occurs in people who engage in fish-related occupations or tropical fish breeders cleaning the fish tank with minor wounds on the upper extremities. In the present case, it is unknown why the lesion occurred in the nasal cavity, but since it developed during the season of Japanese cedar pollinosis, it is possible that the infection occurred from a slight scratch on the nasal vestibular skin and nasal mucosa by the wet hands with contaminated fish tank water, such as from blowing the nose.

Second, IGRA and mycobacterial tissue culture were useful for the diagnosis. When the nasal granuloma is found, it is necessary to distinguish malignant tumors, such as cancer and malignant lymphoma, from benign tumors, such as hemangiomas, polyangiitis granulomatosis, sarcoidosis, and nasal tuberculosis. In this case, we suspected nasal tuberculosis from the positive IGRA test and confirmed the diagnosis by performing tissue culture of mycobacteria. In the 1990s, a gene sequence region of *M. tuberculosis* that was completely lacking in bacillus Calmette–Guérin (BCG) was identified [8]. *M. tuberculosis*-specific proteins such as early secreted antigenic target 6 (ESAT-6), culture filtrate protein 10 (CFP-10), tuberculosis 7.7 (TB 7.7), and others coded in that area were identified. Because IGRA measures the reaction of interferon-gamma with ESAT-6/CFP-10, it is not affected by BCG vaccination and is widely used as a screening test for tuberculosis infection. Most nontuberculous mycobacterial infections are negative for IGRA, but some nontuberculous mycobacteria, including *M. marinum,* may be positive for IGRA [9]. *M. marinum*, like *M. tuberculosis*, possesses a functional ESX-1 (ESAT system-1) secretion system that exports ESAT-6 and CFP-10. Therefore, when IGRA is positive, not only *M. tuberculosis* but also *M. marinum* infection should be considered.

In many cases of *M. marinum* infection, the presence of mycobacteria cannot be confirmed by pathological examination [10–14], and it is highly probable that diagnosis cannot be made unless a mycobacterial tissue culture test is performed. In the present case as well, the acid-fast bacterium could not be identified by multiple histological examinations. This case was diagnosed by a mycobacterial tissue culture test based on the positive result of IGRA, but it took 13 months from the first visit to the diagnosis. The lack of evidence of mycobacteria on histological examination delayed us from suspecting mycobacterial infection. The final diagnosis can only be obtained with mycobacterial culture test.

The number of nontuberculous mycobacterial infections is increasing with the increasing number of patients who use immunosuppressive agents, including anti-TNF biologics [2]. It is necessary to consider the possibility of mycobacterial infection in lesions of the head and neck, especially in patients receiving immunosuppressive drug therapy.

In conclusion, *M. marinum* infection can cause an intranasal mass. IGRA and the mycobacterial tissue culture test are useful for the diagnosis. We must be aware that *M. marinum* infection can manifest as an intranasal mass. Pathological examination of the nasal lesion revealed only an inflammatory nasal granuloma, not mycobacterial infection, and there may be many more “hidden” cases of *M. marinum* infection. In the present case, IGRA was important for diagnosis. IGRA should be more widely used, particularly in patients who are taking immunosuppressive agents; however, the gold standard for diagnosis remains mycobacterial culture. If granulomatous lesions are found in the nasal cavity, it is necessary to perform a mycobacterial tissue culture test for the possibility of mycobacterial infection.

## Data Availability

The primary data about the patient were obtained from the electronic medical record of Tokai University School of Medicine. Cited manuscripts were found on PubMed.

## References

[1] Koh W.-J. (2017). Nontuberculous mycobacteria-overview. *Microbiology Spectrum*.

[2] Henkle E., Winthrop K. L. (2015). Nontuberculous mycobacteria infections in immunosuppressed hosts. *Clinics in Chest Medicine*.

[3] Wentworth A. B., Drage L. A., Wengenack N. L., Wilson J. W., Lohse C. M. (2013). Increased incidence of cutaneous nontuberculous mycobacterial infection, 1980 to 2009: a population-based study. *Mayo Clinic Proceedings*.

[4] Ang P., Rattana-Apiromyakij N., Goh C.-L. (2000). Retrospective study of Mycobacterium marinum skin infections. *International Journal of Dermatology*.

[5] Johnson M. G., Stout J. E. (2015). Twenty-eight cases of Mycobacterium marinum infection: retrospective case series and literature review. *Infection*.

[6] Ho W.-L., Kuo A.-J., Chuang W.-Y., Chan K.-C. (2011). Nasal fish tank granuloma: an uncommon cause for epistaxis. *The American Journal of Tropical Medicine and Hygiene*.

[7] Asakura T., Ishii M., Kikuchi T. (2016). Disseminated Mycobacterium marinum infection with a destructive nasal lesion mimicking extranodal NK/T cell lymphoma. *Medicine*.

[8] Mahairas G. G., Sabo P. J., Hickey M. J., Singh D. C., Stover C. K. (1996). Molecular analysis of genetic differences between mycobacterium bovis BCG and virulent M. bovis. *Journal of Bacteriology*.

[9] Kobashi Y., Mouri K., Yagi S (2009). Clinical evaluation of the QuantiFERON-TB Gold test in patients with non-tuberculous mycobacterial disease. *The International Journal of Tuberculosis and Lung Disease: The Official Journal of the International Union Against Tuberculosis and Lung Disease*.

[10] Holden I. K., Kehrer M., Andersen A. B., Wejse C., Svensson E., Johansen I. S. (2018). Mycobacterium marinum infections in Denmark from 2004 to 2017: a retrospective study of incidence, patient characteristics, treatment regimens and outcome. *Scientific Reports*.

[11] Sia T. Y., Taimur S., Blau D. M. (2015). Clinical and pathological evaluation ofMycobacterium marinumGroup skin infections associated with fish markets in New York city. *Clinical Infectious Diseases*.

[12] Cheung J. P. Y., Fung B., Ip W. Y., Chow S.-P. (2012). Mycobacterium marinum infection of the hand and wrist. *Journal of Orthopaedic Surgery*.

[13] Edelstein H. (1994). Mycobacterium marinum skin infections. Report of 31 cases and review of the literature. *Archives of Internal Medicine*.

[14] Wu T.-S., Chiu C.-H., Yang C.-H. (2012). Fish tank granuloma caused by Mycobacterium marinum. *PLoS One*.

